# Serum cytokine profile in pediatric Sweet’s syndrome: a case report

**DOI:** 10.1186/s13256-017-1317-0

**Published:** 2017-07-02

**Authors:** Yoshihiko Takano, Hisanori Fujino, Akihiro Yachie, Shin-ichi Sumimoto

**Affiliations:** 1 Department of Pediatrics, Sakai City Medical Center, 1-1-1 Ebaraji-Cho, Nishi-Ku, Sakai City, Osaka 593-8304 Japan; 2 Department of Pediatrics, Osaka Red Cross Hospital, 5-30 Fudegasaki-Cho, Tennouji-Ku, Osaka, 543-8555 Japan; 3 Department of Pediatrics, School of Medicine, Institute of Medical, Pharmaceutical, and Health Sciences, Kanazawa University, 13-1 Takaramachi, Kanazawa, Ishikawa 920-8640 Japan

**Keywords:** Case report, Glucocorticoid resistance, Neutrophilic dermatosis, Pro-inflammatory cytokines, Sweet’s syndrome, Urinary tract infections

## Abstract

**Background:**

Sweet’s syndrome is characterized by fever, leukocytosis, and tender erythematous papules or nodules. It is a rare condition, particularly in the pediatric population, and has recently been proposed to be an autoinflammatory disease that occurs due to innate immune system dysfunction, involving several cytokines, which causes abnormally increased inflammation. To the best of our knowledge, no report has documented the cytokine profile in a pediatric patient with Sweet’s syndrome.

**Case presentation:**

A previously healthy 34-month-old Japanese girl was hospitalized because of remittent fever and pain in her right lower extremity with erythematous nodules. A skin biopsy of the eruption revealed dermal perivascular neutrophilic infiltration with no evidence of vasculitis, which led to the diagnosis of Sweet’s syndrome. She was prescribed with orally administered prednisolone and a prompt response was observed; then, the prednisolone dose was tapered. During treatment she developed upper and lower urinary tract infections, after which her cutaneous symptoms failed to improve despite increasing the prednisolone dosage. To avoid long-term use of systemic corticosteroids, orally administered potassium iodide was initiated, but it was unsuccessful. However, orally administered colchicine along with prednisolone effectively ameliorated her symptoms, and prednisolone dosage was reduced again.

We analyzed the circulating levels of interleukin-1β, interleukin-6, interleukin-18, neopterin, and soluble tumor necrosis factor receptors I and II, in order to clarify the pathogenesis of Sweet’s syndrome. Of these cytokines, only interleukin-6 levels were elevated prior to orally administered prednisolone therapy. Following therapy, the elevated interleukin-6 levels gradually diminished to almost normal levels; interleukin-1β and interleukin-18 stayed within normal ranges throughout the treatment. Neopterin became marginally elevated after the start of treatment. Both soluble tumor necrosis factor receptor I and soluble tumor necrosis factor receptor II levels increased shortly after the onset of urinary tract infections.

**Conclusions:**

This is the first case report of pediatric Sweet’s syndrome in which serum cytokine levels were investigated. Future studies should gather more evidence to elucidate the pathophysiology of Sweet’s syndrome.

## Background

Sweet’s syndrome was first described as an “acute febrile neutrophilic dermatosis” in 1964 [1], which clinically manifests with symptoms of fever, leukocytosis, and tender, well-demarcated erythematous papules or nodules. Usually, Sweet’s syndrome is diagnosed based on the criteria proposed by von den Driesch in 1994 (Table 1) [2] and is clinically classified into three categories: idiopathic, malignancy-associated, and drug-induced [3]. Sweet’s syndrome mainly affects middle-aged women and pediatric cases of Sweet’s syndrome are so rare that, as of 2014, fewer than 100 cases have been reported [4].

**Table 1:** Diagnostic criteria for Sweet’s syndrome [2]

Major criteria are as follows:
• Abrupt onset of tender or painful erythematous plaques or nodules, occasionally with vesicles, pustules, or bullae
• Predominantly neutrophilic infiltration in the dermis without leukocytoclastic vasculitis
Minor criteria are as follows:
• Preceding nonspecific respiratory or gastrointestinal tract infection, or vaccination, or associated with inflammatory disease, hemoproliferative disorders, solid malignant tumors, or pregnancy
• Periods of general malaise and fever (body temperature >38 °C)
• Meeting three out of the following four laboratory values during onset is necessary; 1) an erythrocyte sedimentation rate >20 mm, 2) positive C-reactive protein (CRP) result, 3) segmented nuclear neutrophils, bands >70% in peripheral blood smears, and 4) leukocytosis (count >8000/μL)
• Excellent response to treatment with systemic corticosteroids or potassium iodide

Both major and two minor criteria are needed for diagnosis

Recently, Sweet’s syndrome has been suggested to be an autoinflammatory disease. Such diseases are characterized by abnormally increased inflammation due to innate immune system dysfunction, in which several cytokines play pivotal roles in provoking neutrophil-mediated inflammation [5]. Although cytokines in adult cases of Sweet’s syndrome have been assessed by some researchers, to the best of our knowledge, there is no report that has documented the cytokine profile in a pediatric patient with Sweet’s syndrome. In this report, we present a case of a pediatric patient with Sweet’s syndrome in whom serum cytokine levels were investigated.

## Case presentation

**Fig. 1:**
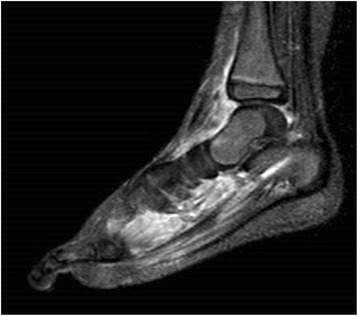
Magnetic resonance imaging of the right foot using short-tau inversion-recovery sequence revealed high signal intensity (*white*) in the soft tissue from the ankle to toe, indicating inflammatory changes

**Fig. 2:**
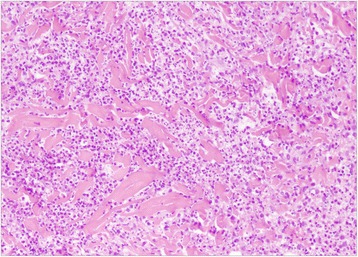
Histology of the skin lesion (hematoxylin-eosin stain, original magnification × 200). Skin biopsy revealing dense neutrophilic infiltration without signs of vasculitis

A 34-month-old Japanese girl presented with a history of 7 days of remittent fever and 1 month of pain in her right lower leg. She was born to non-consanguineous, healthy parents and had no remarkable previous medical history, family history, medication, allergy, recent vaccination, or antecedent infection. A physical examination revealed no abnormality except for tender erythematous nodules in her right lower extremity. Initial laboratory studies indicated intense inflammation evidenced by a C-reactive protein (CRP) level of 121 mg/L, erythrocyte sedimentation rate (ESR) of >140 mm/hour, with a white blood cell count of 13,500/mm^3^. An extensive evaluation of serum markers for infectious, autoimmune, or malignant disease failed to determine the etiology. Despite negative culture results, we empirically administered antibiotics; however, they appeared unsuccessful. Five weeks after the first visit, her right sole became severely swollen with an expanding eruption, and laboratory studies revealed a remarkably augmented inflammatory pattern (CRP 177 mg/L, ESR >160 mm/hour) with aggravated leukocytosis (20,530/mm^3^). Further investigation which included bone marrow aspiration, whole body gallium scintigraphy, and ophthalmological examination revealed no abnormality. However, magnetic resonance imaging revealed inflammatory changes in the soft tissue from her ankle to her sole, suggesting panniculitis (Fig. 1). Biopsy of the eruption demonstrated dermal perivascular neutrophilic infiltration with no evidence of vasculitis (Fig. 2). The clinical features met the criteria for Sweet’s syndrome and a diagnosis was made. Following diagnosis, she was started on orally administered prednisolone (PSL 2 mg/kg per day) which was a first-line treatment for Sweet’s syndrome. Her fever and leg tenderness subsided promptly, so the dose of PSL was reduced to 1 mg/kg per day. While on PSL, she developed upper, then lower, urinary tract infections (UTIs). Therefore, we administered antibiotics for 3 weeks, however, neither clinical symptoms nor laboratory results improved. We increased the PSL dosage to 2 mg/kg per day, and tried to determine the presence of any underlying condition; however, all investigations showed normal results. To avoid long-term use of systemic corticosteroids, we tried other first-line treatments. Orally administered potassium iodide (30 mg/kg per day) was initiated, but this was unsuccessful; however, orally administered colchicine (0.03 mg/kg per day) combined with PSL (2 mg/kg per day) effectively ameliorated her symptoms, and her CRP levels almost normalized. Tapering of PSL dose was being attempted at the time of writing this report.

Cytokine analysis of the remaining frozen serum specimens demonstrated that interleukin (IL)-6 levels, that were elevated prior to therapy, gradually decreased to near-normal levels. In contrast, levels of IL-1β, IL-18, neopterin, and soluble tumor necrosis factor receptors (sTNF-R) I and II were not elevated before treatment. Of these cytokines, IL-1β and IL-18 stayed within their normal ranges throughout the treatment, while neopterin became marginally elevated after the start of treatment. Both sTNF-RI and sTNF-RII levels became elevated shortly after the onset of UTI (Table 2).

**Table 2:** Changes in medical therapy and cytokine levels over 10 weeks

Treatment	Reference range [6, 7]	Pre	1 week	◆	2 weeks	4 weeks	5 weeks	8.5 weeks	10 weeks
PSL (mg/kg per day)		-	2	1		1	2	2	1
Colchicine		-	-	-		-	-	+	+
Antibiotics		-	-	⇒ ⇒ ⇒⇒		⇒ ⇒ ⇒	-	-	-
IL-1β (pg/mL)	<3.2	<0.12	---		---	---	0.66	0.13	0.22
IL-6 (pg/mL)	<5	48	20		---	20	5	8	4
IL-18 (pg/mL)	<500	200	255		---	215	280	193	184
Neopterin (nmol/L)	<5	4.1	5.2		---	5.9	5.1	5.3	7.1
sTNF-RI (pg/mL)	484–1407	1020	1180		---	1860	2320	1150	1260
sTNF-RII (pg/mL)	829–2262	1260	1930		---	2880	3200	1400	2730
CRP (mg/L)	<4	102	23		54	68	8	4	44
ESR (mm/hour)	<10	>160	8		29	29	6	---	7
WBC (/mm^3^)	5800–16,300	16,170	32,500		35,240	24,730	26,080	21,480	13,860

*CRP* C-reactive protein, *ESR* erythrocyte sedimentation rate, *IL* interleukin, *PSL* prednisolone, *sTNF-RI* soluble tumor necrosis factor receptor I, *sTNF-RII* soluble tumor necrosis factor receptor II, *WBC* white blood cell

The symbol ‘◆’ represents the onset of the antibiotic therapy performed for urinary tract infections that developed one week after PSL initiation

The symbol ‘⇒’ represents antibiotic therapy for UTI is being performed

The values of pre-treatment are the results just before PSL initiation, and differ from the ones before diagnosis

5 weeks means 5 weeks from the initiation of PSL treatment

## Discussion

This is the first report in which serum cytokine levels were investigated in a pediatric patient with Sweet’s syndrome. Because of the excellent response to the initial administration of corticosteroids, which is also used as a diagnostic criterion [2], we are confident in our diagnosis of this case as an idiopathic case of Sweet’s syndrome, as no other causative etiology could be found. However, long-time close monitoring and repeated investigations at appropriate intervals are necessary, because an unknown underlying disease may manifest long after initial presentation.

To elucidate the pathogenesis of Sweet’s syndrome from the perspective of cytokine profile, we investigated the circulating cytokine levels. We hypothesized that if changes in cytokine levels showed an association with disease activity, the cytokine profile could help pave the way for developing specific cytokine blockade therapy. Such therapy could replace the current nonspecific immunosuppressive therapies used, such as systemic corticosteroid administration, which is currently the first-line treatment for Sweet’s syndrome, in which relapses are commonly seen and long-term treatment is usually needed [2].

Because the age-related reference range of serum cytokine levels is not established for all the cytokines employed in our study, we used data from peer-reviewed, published articles as a reference. We adopted an article by Kleiner *et al*. [6] for the reference range of serum IL-1β levels, and another one by Shimizu *et al*. [7] for the rest of the cytokines.

Contrary to a previous study where topical cytokines were investigated [8], serum levels of IL-1β, which is considered to be a key cytokine in autoinflammatory diseases [9], were not elevated in our case. However, this result does not mean that Sweet’s syndrome is excluded from autoinflammatory diseases, because some autoinflammatory diseases do not have an apparent relationship to IL-1 [5].

In our case, serum levels of IL-6, a major pro-inflammatory cytokine, decreased to some extent with initial PSL administration, but were not completely normalized during treatment for the UTIs, which seemed to be correlated with poor responsiveness to PSL. We think that this observation might be associated with glucocorticoid resistance (GCR) that means poor or low responsiveness to glucocorticoids. Although the exact mechanism of GCR remains unknown, it reportedly consists of multiple processes involving several pro-inflammatory cytokines including IL-1β, IL-6, and tumor necrosis factor-α (TNF-α) [10]. In our case, the responsiveness to corticosteroid therapy appeared to lessen after the development of UTIs, which could elevate serum pro-inflammatory cytokines [11, 12]. Therefore, the UTIs observed in our case might lead to GCR through this mechanism, although causality has not yet been established. This highlights the importance of taking strong precautions to avoid infections during systemic corticosteroid therapy.

IL-18 is another pro-inflammatory cytokine closely related to IL-1β, which enhances interferon-γ (IFN-γ) secretion [9]; serum neopterin level is a marker of cellular immunity activated mainly by IFN-γ [13]. In our case, serum IL-18 levels stayed within the normal range, while neopterin levels became marginally elevated after the start of treatment, although the significance of this elevation is unclear. These observations suggest that IFN-γ may be secreted by mechanisms other than IL-18 stimulation.

Serum levels of sTNF-RI/RII are considered to reflect the activity of TNF-α function [14]. Within this framework, TNF-α does not seem to exert its function before the onset of UTIs.

We found four previous reports in which serum cytokine levels of adult patients with Sweet’s syndrome are described [15–18]. In the adult population, Sweet’s syndrome is often associated with malignant disease, especially myelodysplastic syndrome; hence, cytokine profiles of adult patients with Sweet’s syndrome might differ from those of their pediatric counterparts. However, in three of the four articles, serum IL-6 levels were reported to be elevated similarly to that in our case, while other cytokines did not show definite patterns (Table 3).

**Table 3:** Serum cytokine levels in adult patients with Sweet’s syndrome

	IL-1β	IL-6	TNF	IFN-γ/neopterin	Other cytokines	Patients (underlying disease)
Reuss-Borst *et al*. [15]	→	↑	→ ~ ↑	→	G-CSF ↑	Adult female patient with SS (with MDS)
Loraas *et al*. [16]	---	↑	→	---	G-CSF →, TNF-β →	Adult male patient with SS (with MDS)
Giasuddin *et al*. [17]	↑	---	---	↑	IL-2 ↑, IL-4 →	8 adult female patients with SS
Hattori *et al*. [18]	→	↑	---	---	G-CSF ↑	Adult male patient with SS (with MDS)
Our case	→	↑	→ ~ ↑	→ ~ ↑	IL-18 →	34-month-old female with idiopathic SS

*G-CSF* granulocyte-colony stimulating factor, *IFN* interferon, *IL* interleukin, *MDS* myelodysplastic syndrome, *SS* Sweet’s syndrome, *TNF* tumor necrosis factor

The symbol → ~ ↑ means normal or slightly elevated value, The symbol → means normal elevated value, The symbol↑ means slightly elevated value

Since Sweet’s syndrome is a heterogeneous entity, we believed that the cytokine profile might differ depending on the underlying disease; however, there might be a certain trend for IL-6 elevation, although we did not find its significance in the pathophysiology of Sweet’s syndrome.

As this study was limited to a single case report, we could not provide conclusive insights into the pathogenesis of Sweet’s syndrome. However, this study suggests that cytokine analysis is an important aspect, and may help elucidate the pathogenesis of Sweet’s syndrome and other inflammatory diseases.

## Conclusions

This is the first case report to our knowledge of pediatric Sweet’s syndrome in which serum cytokine levels were investigated. At present, in terms of serum cytokine levels, there is not sufficient evidence to elucidate the pathophysiology of Sweet’s syndrome.

## Abbreviations

CRP: C-reactive protein
ESR: Erythrocyte sedimentation rate
GCR: Glucocorticoid resistance
IFN-γ: Interferon-gamma
IL: Interleukin
PSL: Prednisolone
sTNF-R: Soluble tumor necrosis factor receptor
TNF-α: Tumor necrosis factor-alpha
UTI: Urinary tract infection
